# Arterial stiffness & Sri Lankan chronic kidney disease of unknown origin

**DOI:** 10.1038/srep32599

**Published:** 2016-09-02

**Authors:** Fiona Gifford, Robert Kimmitt, Chula Herath, David J Webb, Vanessa Melville, Sisira Siribaddana, Michael Eddleston, Neeraj Dhaun

**Affiliations:** 1 University/British Heart Foundation Centre of Research Excellence, University of Edinburgh, The Queen’s Medical Research Institute, 47 Little France Crescent, Edinburgh, EH16 4TJ, UK; 2 South Asian Clinical Toxicology Research Collaboration (SACTRC), University of Peradeniya, UK; 3 Sri Jayewardenepura General Hospital, Nugegoda, Sri Lanka; 4 Faculty of Medicine and Applied Sciences, Rajarata University of Sri Lanka; 5 Department of Renal Medicine, Royal Infirmary of Edinburgh, UK

## Abstract

Chronic kidney disease (CKD) is common and independently associated with cardiovascular disease (CVD). Arterial stiffness contributes to CVD risk in CKD. In many developing countries a considerable proportion of CKD remains unexplained, termed CKD*u*. We assessed arterial stiffness in subjects with Sri Lankan CKD*u*, in matched controls without CKD and in those with defined CKD. Aortic blood pressure (BP), pulse wave velocity (PWV) and augmentation index (AIx) were assessed in 130 subjects (50 with CKD*u*, 45 with CKD and 35 without CKD) using the validated TensioMed™ Arteriograph monitor. Brachial and aortic BP was lower in controls than in CKD*u* and CKD subjects but no different between CKD*u* and CKD. Controls had a lower PWV compared to subjects with CKD*u* and CKD. Despite equivalent BP and renal dysfunction, CKD*u* subjects had a lower PWV than those with CKD (8.7 ± 1.5 *vs.* 9.9 ± 2.2 m/s, p < 0.01). Excluding diabetes accentuated the differences in PWV seen between groups (controls *vs.* CKD*u vs.* CKD: 6.7 ± 0.9 *vs.* 8.7 ± 1.5 *vs.* 10.4 ± 1.5 m/s, p < 0.001 for all). Sri Lankan CKD*u* is associated with less arterial stiffening than defined causes of CKD. Whether this translates to lower cardiovascular morbidity and mortality long term is unclear and should be the focus of future studies.

Chronic kidney disease (CKD) is a significant cause of morbidity and mortality worldwide^1^. In many developing countries a considerable proportion of CKD remains unexplained by traditional risk factors. This is often called chronic kidney disease of unknown etiology (CKD*u*). CKD*u* is a diagnosis of exclusion, made when a patient fulfills the Kidney Disease Improving Global Outcomes (KDIGO) CKD criteria but without evidence of a recognized cause such as diabetes, hypertension, or glomerulonephritis^2^. Over the last decade CKD*u* has become a leading cause of death in Sri Lanka^3^.

CKD is an independent risk factor for cardiovascular disease (CVD)^4^. Increased arterial stiffness, as measured by pulse wave velocity (PWV), is a characteristic feature of CKD^5^, a recognised marker of CVD risk^5^,^6^, and an independent predictor of mortality and survival in CKD^6^,^7^. To date, there have been no studies of arterial stiffness in those with pre-dialysis CKD*u*. Furthermore, it remains unclear whether the CVD risk associated with CKD*u* is the same as that of CKD of known cause. We hypothesised that CKD*u* patients would have increased arterial stiffness compared to matched controls but that these measures would be no different compared to matched subjects with defined aetiologies for their CKD.

## Materials & Methods

This was a prospective, cross-sectional case controlled study performed in Anuradhapura, North Central Province and Colombo, Sri Lanka with approval of the Ethics Review Committee, Faculty of Medicine & Allied Sciences, Rajarata University of Sri Lanka and the written informed consent of each subject. The investigations conformed to the principles outlined in the Declaration of Helsinki.

### Subjects

Subjects were aged between 18 and 85 years. Chronic kidney disease of unknown origin (CKD*u*) was defined as evidence of renal dysfunction (proteinuria or Chronic Kidney Disease Epidemiology Collaboration (CKD-EPI) eGFR <60 ml/min/1.73 m^2^) with no clear aetiological factor (history of diabetes mellitus, long-standing hypertension–defined as a BP ≥160/100 mmHg untreated or ≥140/90 mmHg whilst taking antihypertensive medication, history of known renal disease). The diagnosis of CKD was based on the same criteria but with an established cause for the renal impairment. A group of age- and sex-matched controls were also recruited from the same geographical area.

### Study protocol

Following a 10 min rest period measurements of BP and arterial stiffness were taken with the TensioMed™ Arteriograph monitor (TensioMed Ltd, Hungary). Measurements were taken as the average of two readings. The Arteriograph utilises brachial pulse oscillations to calculate aortic pulse wave velocity (PWV), augmentation index (AIx), systolic BP and pulse pressure. It also records brachial systolic BP and diastolic BP. The Arteriograph has been validated against an invasive measure of aortic PWV^8^ and two commonly used non-invasive measurement devices, the Complior and SphygmoCor^9^,^10^.

Blood was sampled routinely for serum creatinine. Creatinine clearance, as an estimate of GFR, was calculated according to the CKD-EPI equation^11^.

### Statistical analysis

There are no previous studies of pre-dialysis CKD*u* with which we could perform a power calculation. Thus, based on data from previous studies in western populations^12^,^13^ we aimed to recruit at least 30 subjects into each of the 3 groups. Data were stored and statistical analysis performed using Graph Pad Prism, version 6.0 (Graph Pad Software Inc. San Diego, California). Data are presented as mean ± standard deviation (SD). Differences between groups were assessed using ordinary one-way ANOVA with Tukey’s multiple comparisons post-hoc test. Statistical significance was taken at the 5% level.

## Results

**Figure 1:**
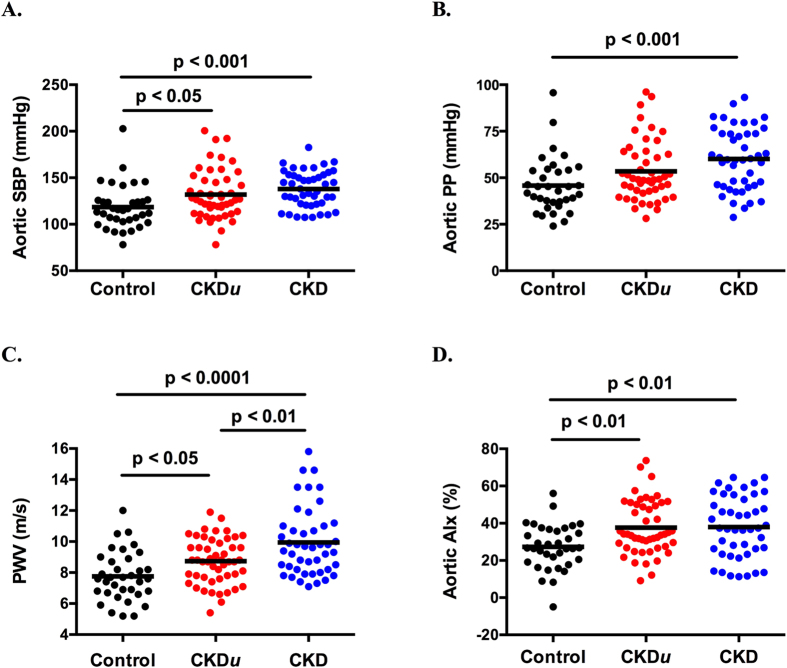
Aortic systolic BP (SBP) (**A**), aortic pulse pressure (PP) (**B**), aortic pulse wave velocity (PWV) (**C**) and aortic augmentation index (AIx) (**D**) in control subjects, those with CKD*u* and CKD. Horizontal black line represents mean. Ordinary one-way ANOVA with Tukey’s multiple comparisons test was used to compare all groups.

Subject characteristics are shown in Table 1. The 3 groups were well matched. However, there were fewer smokers in both CKD groups than in the control group, and those with CKD had a modestly higher body mass index (BMI) than those with CKD*u*. As expected, brachial and aortic blood pressure (BP) was lower in controls than in CKD*u* and CKD subjects. Importantly, CKD*u* and CKD subjects did not differ in terms of brachial or aortic BP components (Fig. 1A,B). Interestingly, in the face of equivalent BP and degree of renal dysfunction, those with CKD*u* had a ~15% lower PWV than those with CKD (Fig. 1C,D). Aortic augmentation index (AIx) was no different between the 2 groups.

**Figure 2:**
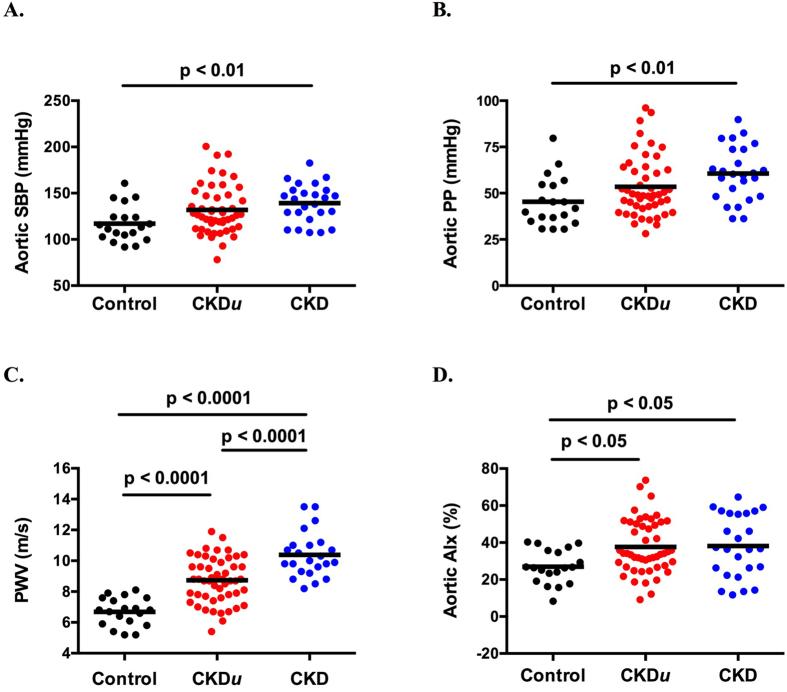
Aortic systolic BP (SBP) (**A**), aortic pulse pressure (PP) (**B**), aortic pulse wave velocity (PWV) (**C**) and aortic augmentation index (AIx) (**D**) in control subjects, those with CKDu and CKD, excluding subjects with diabetes. Horizontal black line represents mean. Ordinary one-way ANOVA with Tukey’s multiple comparisons test was used to compare all groups.

Hypertension and diabetes are both important contributors to arterial stiffness^14^. Although the number of subjects with hypertension was similar in the CKD*u* and CKD groups, diabetes was only present in those with CKD. Thus, we re-analysed our measures of arterial stiffness excluding those with diabetes. This did not affect differences between groups in terms of age, BMI, BP, degree of renal dysfunction or prevalence of hypertension (Table 2 and Fig. 2A,B). Importantly, the absence of diabetes accentuated the differences in PWV seen between groups – subjects with CKD*u* had a ~30% higher PWV than matched controls; PWV in those with CKD was ~20% higher than that associated with CKD*u* (Fig. 2C,D).

## Discussion

To our knowledge this is the first study exploring arterial stiffness in a population of Sri Lankan subjects with CKD*u*. In keeping with other studies from developed countries we have found that those with CKD have a higher PWV than those without CKD^15^. This is important because PWV is an established independent predictor of mortality and survival in CKD^6^,^7^ and is also modifiable^16^. Our control group, who had an estimated glomerular filtration rate (eGFR) >60 ml/min/1.73 m^2^, had comparable PWV values to those seen in other similar cohorts both in the presence^12^ and absence^13^ of diabetes.

Most noteworthy was our finding that, despite similar eGFR and BP, those with CKD*u* had a lower PWV than those with a defined cause for CKD. CKD*u* is primarily a disorder of the tubulointerstitium whereas most causes of CKD primarily affect the glomeruli (IgA nephropathy, diabetes, hypertension). A few studies^17^ suggest arterial stiffness in CKD may vary by underlying etiology and our data add to these. Furthermore, one study in subjects with end-stage renal failure due to Balkan nephropathy (an endemic nephropathy similar to Sri Lankan CKD*u*), investigated arterial stiffness using the same technique as here, and found that PWV was lower than in those with other causes for their dialysis-requiring renal failure^18^. The authors suggested the difference may be due to the later occurrence of hypertension in those with Balkan nephropathy, which is also a feature of CKD*u* in Sri Lanka^3^.

Although PWV varied significantly between CKD and CKD*u* we found no difference in AIx. Whilst PWV and AIx are both used as surrogate measures of arterial stiffness they are not interchangeable. PWV is an assessment of large artery stiffness. AIx is derived from analysis of the pulse contour and is the proportion of central pulse pressure that results from arterial wave reflection. Although the timing of the arrival of the reflected wave at the proximal aorta is largely determined by large artery PWV, AIx is not simply a surrogate measure of PWV. It is influenced by vasoactive drugs independently of PWV^19^, suggesting that it is also determined by the intensity of wave reflection which, in turn, is determined by the diameter and elasticity of small arteries and arterioles. AIx increases with mean arterial pressure^20^ and is inversely related to heart rate^21^ and body height^22^, so these variables should be accounted for when interpreting studies that use pulse contour analysis. In the current study some of these factors may account for the lack of difference in AIx between CKD and CKD*u* as could under-powering of the study with respect to this parameter.

Although this is the first study of its kind we recognise a number of limitations. Our study size increases the chances of our results being subject to a type II error. Larger similar studies are needed to confirm our findings. Furthermore, some of the medications taken by our patients, such as ACE inhibitors, angiotensin receptor blockers, and ß blockers may have affected arterial stiffness. However, all patients were stabilised on their therapies and the CKD groups were on similar treatments in this respect. We also saw a high prevalence of diabetes in our cohort and so our findings may not be generalizable to population outside Sri Lanka. Finally, we were unable to assess the influence of proteinuria or other more novel CVD risk factors such as circulating C-reactive protein, asymmetric dimethylarginine or endothelin-1, all of which may contribute to arterial stiffening^23^ and so may, in part, explain the differences we are seeing. This was not possible in the current study but should be the focus for future work.

### Significance

Measures of arterial stiffness are not currently used in the clinic. However, there have been a number of clinical studies that have examined differences in PWV between health and disease. We have previously shown that patients with CKD stage IV – the mean eGFR in the current study fits into this–had a PWV of 7.0 ± 1.4 m/s compared to 6.2 ± 0.9 m/s in matched controls. This ~13% is difference similar to the magnitude of the difference observed here between CKD and CKD*u*. More importantly, a few clinical trials have demonstrated that differential lowering of PWV with medical treatment results in different cardiovascular or renal outcomes^24^,^25^ and the importance of such studies is underscored by epidemiological data that suggest that PWV is an independent risk factor for cardiovascular morbidity and mortality^6^,^14^,^26^. Karalliedde *et al*. have previously shown that treatment of patients with type 2 diabetes and proteinuria with 6 months of valsartan/hydrochlorthiazide reduced PWV by ~14% (12.5 to 10.7 m/s)^27^. In the same study the calcium channel blocker amlodipine reduced PWV by ~6%, a similar effect in magnitude to that seen with endothelin receptor antagonists^16^.

This is the first study suggesting that Sri Lankan CKD*u* is associated with less arterial stiffening than other defined causes of CKD. There remain a number of unanswered questions that should be the focus of future work: what is the natural history of arterial stiffness in CKD*u*; is this modifiable; and, if so, does this translate to lower cardiovascular morbidity and mortality long term compared to other forms of CKD.

## Additional Information

**How to cite this article**: Gifford, F. *et al*. Arterial stiffness & Sri Lankan chronic kidney disease of unknown origin. *Sci. Rep.* **6**, 32599; doi: 10.1038/srep32599 (2016).

## Figures and Tables

**Table 1:** Study participant data–all subjects.

Parameter	Controls (n = 35)	CKD*u* (n = 50)	CKD (n = 45)
*Demographic*			
Age, years	56 ± 9	58 ± 10	61 ± 11
Male sex, n (%)	24 (69)	35 (70)	34 (76)
Current smoker (%)	17 (49)	6 (12)	4 (8)
*Clinical*			
BMI, kg/m^2^	22 ± 5	22 ± 4	24 ± 4**
Brachial blood pressure, mmHg			
Systolic	121 ± 20*	131 ± 22	135 ± 15
Diastolic	73 ± 11*	79 ± 12	81 ± 11
Creatinine, μmol/L	77 ± 13****	434 ± 380	292 ± 143
CKD stage			
1	—	0	0
2	—	0	0
3	—	17	17
4	—	11	16
5	—	22	12
Estimated GFR, mL/min /1.73m^2^	89 ± 16****	23 ± 17	24 ± 13
Hypertension (%)	18 (51)	34 (68)	31 (69)
Diabetes mellitus (%)	16 (46)	0 (0)	22 (49)
*Medications, n (%)*			
α-blocker	2 (6)	8 (16)	7 (16)
ACE inhibitor	8 (23)	32 (64)	29 (64)
ARB	3 (9)	36 (72)	32 (71)
β-blocker	7 (20)	20 (40)	18 (40)
Calcium channel blocker	6 (17)	18 (36)	21 (47)
Diuretic	2 (6)	31 (62)	27 (69)
Statin	16 (46)	0 (0)	22 (49)

Values are given as mean ± SD (%). ACE: angiotensin converting enzyme; ARB: angiotensin receptor blocker; BMI: body mass index; CKD: chronic kidney disease; CKD*u*: chronic kidney disease of unknown origin; DBP: diastolic BP; eGFR: estimated glomerular filtration rate; SBP: systolic BP. Ordinary one-way ANOVA with Tukey’s multiple comparisons test was used to compare baseline characteristics between all groups.^*^p < 0.05 *vs.* CKD*u* and *vs.* CKD, ^**^p < 0.01 *vs.* CKD*u*, ^****^p < 0.0001 *vs.* CKD*u* and *vs.* CKD.

**Table 2:** Study participant data – subjects with diabetes excluded.

Parameter	Controls(n = 19)	CKD*u*(n = 50)	CKD(n = 23)
*Demographic*			
Age, years	54 ± 10*	58 ± 10	62 ± 12
Male sex, n (%)	13 (68)	35 (70)	16 (70)
Current smoker (%)	10 (53)	6 (12)	0 (0)
*Clinical*			
BMI, kg/m^2^	22 ± 4	22 ± 4	25 ± 4**
Brachial blood pressure, mmHg			
Systolic	120 ± 16*	131 ± 22	136 ± 16
Diastolic	72 ± 9*	79 ± 12	81 ± 14
Creatinine, μmol/L	76 ± 16****	434 ± 380	287 ± 132
Estimated GFR, mL/min /1.73m^2^	91 ± 19****	23 ± 17	24 ± 13
Hypertension (%)	8 (42)	34 (68)	13 (57)

Values are given as mean ± SD (%). BMI: body mass index; CKD: chronic kidney disease; CKD*u*: chronic kidney disease of unknown origin; DBP: diastolic BP; eGFR: estimated glomerular filtration rate; SBP: systolic BP. Ordinary one-way ANOVA with Tukey’s multiple comparisons test was used to compare baseline characteristics between all groups.*p < 0.05 vs. CKD, **p < 0.01 vs. CKD*u*, ****p < 0.0001 vs. CKD*u* and vs. CKD.
